# Brain imaging-derived phenotypes influence risk of bipolar disorder: comprehensive exposome-wide association and wide-angled genetic analyses

**DOI:** 10.1186/s12916-026-05152-9

**Published:** 2026-08-17

**Authors:** Wenzhuo Yang, Lin Pan, Haoqun Xie, Jialin Deng, Yifan Li, Yu Hu, Yihe Zhang, Kaijun Sun, Hongyu Chen, Laiyu Yang, Zijian Chen, Bincheng Yu, Leyan Chen, Zibing Zhao, Yonggao Mou, Yinsheng Chen, Sheng Zhong

**Affiliations:** 1 Department of Neurosurgery, State Key Laboratory of Oncology in South China, Collaborative Innovation Center for Cancer Medicine, Sun Yat-sen University Cancer Center, Guangzhou, China; 2 Department of Neurology, Neuroscience Research Center, The First Hospital of Jilin University, Changchun, China; 3 ZhongShan School of Medicine, Sun Yat-Sen University, 74 Zhongshan Road II, Guangzhou, China; 4 School of Psychology, Center for Studies of Psychological Application, Guangdong Key Laboratory of Mental Health and Cognitive Science, Ministry of Education Key Laboratory of Brain Cognition and Educational Science, South China Normal University, Guangzhou, China; 5 Department of Health Outcomes & Biomedical Informatics, University of Florida, Gainesville, FL USA; 6 China-Japan Union Hospital of Jilin University, Jilin University Changchun, 130033 China

**Keywords:** Bipolar disorder (BD), Brain imaging-derived phenotypes (IDPs), Exposome-wide association study (ExWAS), Neuroimaging biomarkers, Genetic correlation

## Abstract

**Background:**

Despite many reported imaging markers for bipolar disorder (BD), systematic assessment across diverse brain imaging-derived phenotypes (IDPs) is lacking. We used epidemiological and genetic analyses to progress from association to causality and identify candidate biomarkers and potential pathways of BD.

**Methods:**

We conducted an Exposome-Wide Association Study (ExWAS) in 35,680 UK Biobank participants to assess associations of 686 brain IDPs with BD. Genetic correlations were estimated via linkage disequilibrium score regression (LDSC). Bidirectional two-sample Mendelian randomization (MR) and meta-analysis were performed using large-scale GWAS data to infer causality. Additional analyses, including Functional Mapping and Annotation (FUMA), protein-protein interaction (PPI) network analysis, and spatiotemporal gene expression profiling during human brain development, were performed to investigate potential biological mechanisms of BD and IDPs.

**Results:**

In our ExWAS analyses, we identified 10 significant exposome-level IDPs, and LDSC further revealed 67 IDPs with significant genetic correlations with BD. The MR analyses provided robust causal evidence for 12 IDPs, 6 of which were further validated by meta-analysis. Integrative analyses identified two associations supported by both epidemiological and genetic evidence (IDP.1510: fractional anisotropy in the forceps major, and IDP.0387: volume of the right lateral orbital frontal cortex). Additionally, we identified pleiotropic genes between IDPs and BD. Gene enrichment analyses implicated shared biological pathways, such as nucleosome assembly and neutrophil extracellular trap formation . PPI network analysis suggested that histone family members occupied central hubs in the interaction network. Spatiotemporal expression profiling identified region-specific developmental trajectories of pleiotropic genes in brain regions, including the cerebellum and thalamus, with the late fetal to early postnatal period potentially representing a sensitive developmental period for neurodevelopmental effects of BD susceptibility genes.

**Conclusions:**

This study establishes an atlas of brain IDPs associated with BD, by integrating epidemiological and genetic evidence. The findings highlight candidate IDPs, including right lateral orbital frontal cortex volume and fractional anisotropy in the forceps major, that were consistently associated with BD across complementary analyses. Our results provide genetically supported targets for early intervention and precision psychiatry.

## Background

Bipolar disorder (BD) affects roughly 40 million individuals worldwide, with a prevalence of up to 2% in the United States [1, 2]. Patients with BD can exhibit varying degrees of emotional disturbance and cognitive impairment, which has already been listed by the World Health Organization (WHO) as one of the top 20 leading medical causes of disability [3]. Despite its substantial impact, the etiology of BD remains largely unknown, and effective clinical management strategies are limited [4]. Identifying reliable early biomarkers for BD and elucidating its biological mechanisms are critical for advancing diagnosis, treatment, and prevention strategies.

Brain imaging-derived phenotypes (IDPs), measured non-invasively using magnetic resonance imaging (MRI), could potentiate early diagnosis of brain disorders. In recent years, utilizing various neuroimaging methods such as diffusion tensor imaging (DTI), diffusion kurtosis imaging (DKI) and others, researchers have demonstrated that there are many significant alterations in the brain structure in patients with BD compared to healthy individuals [5–7]. For example, Sung Woo Joo et al. confirmed that fractional anisotropy (FA) is lower in corticothalamic white matter fibers in patients with BD, whereas radial diffusivity (RD) is higher in the left external capsule than in healthy controls [8]. However, most studies examining IDP-BD associations have focused on a limited subset of IDPs and lack systematic or comprehensive investigation. Furthermore, the extent to which these associations reflect shared genetic architecture or underlying biological mechanisms remains unclear.

The exposome-wide association study (ExWAS) approach enables systematic evaluation of associations between numerous exposures and health outcomes [9]. Similar to genome-wide association studies (GWAS), which assess associations between multiple genetic factors and health outcomes, ExWAS is an untargeted approach that identifies potential associations for further investigation while accounting for multiple hypothesis testing. Over the past decade, ExWAS has been used to elucidate relationships between environmental exposures and health outcomes, including brain health and cognitive function [10, 11]. However, its application to brain IDPs in the context of BD remains limited.

Several genetic studies have identified variants associated with IDPs. For example, Elliott et al. used GWAS to identify genetic variants linked to MRI markers of brain disorders [12]. The increasing number of published GWAS has led to the development of methods for detecting cross-trait shared genetic architecture using GWAS summary statistics. The linkage disequilibrium score regression (LDSC) statistic estimates single-nucleotide polymorphism (SNP)-based genetic correlations between traits [13]. Mendelian randomization (MR) has been proposed to assess putative causal relationships between traits by using genetic variants as instruments, which mimics random allocation in randomized controlled trials and reduces confounding [14]. Gene-level analyses, such as Multi-marker Analysis of GenoMic Annotation (MAGMA) and Pleiotropic Analysis under Composite Null Hypothesis (PLACO), as well as protein-protein interaction (PPI) network analysis and spatiotemporal gene expression profiling, can identify shared susceptibility genes, relevant pathways, and developmental contexts linking complex traits. These approaches have advanced the understanding of shared genetic architecture across complex phenotypes and diseases [15–17]. However, few studies to date have identified causal relationships between IDPs and BD, and their underlying mechanisms remain largely unclear.

**Fig. 1:**
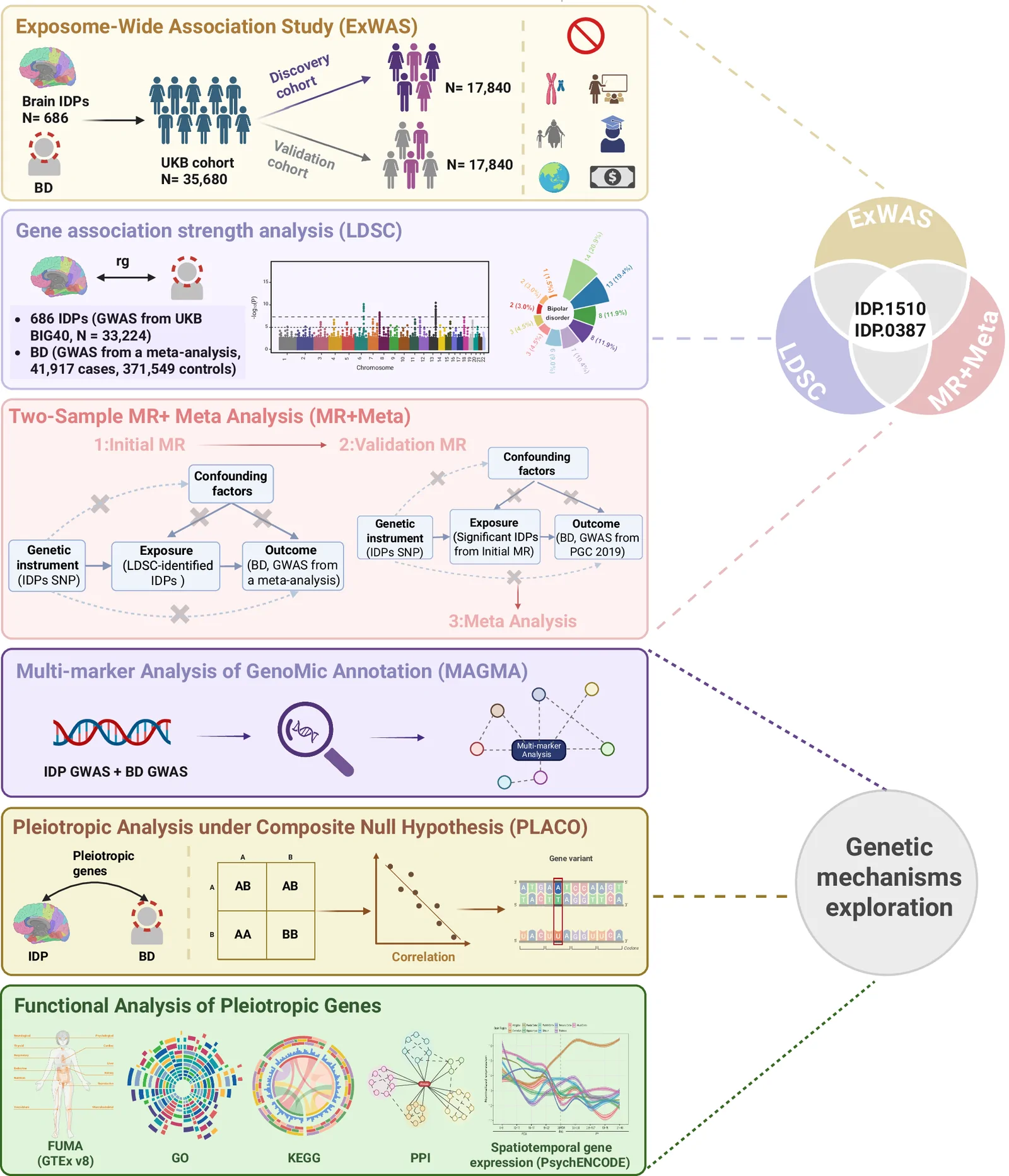
The flow chart of this study

In our study, we implemented a systematic, multi-stage analytical framework to dissect the complex relationship between brain IDPs and BD based on a large cohort of 35,680 individuals (Fig. 1). We began with the ExWAS across 686 brain IDPs in a large-scale cohort to identify robust observational associations. For IDPs showing significant associations, we quantified their genetic correlations with BD using LDSC. Next, we applied bidirectional two-sample MR to rigorously assess causality, leveraging summary statistics from the largest available GWAS datasets, including those from the Psychiatric Genomics Consortium (PGC). This multi-step analytical framework-progressing from broad association screening to targeted causal inference-was further strengthened by sensitivity analyses to ensure robustness. Finally, in order to explore the potential biological mechanisms of these relationships, we employed MAGMA, PLACO, FUMA, PPI network analysis, as well as spatiotemporal gene expression landscape across human brain development. These methods were used to identify potentially relevant genes and pathways that may contribute to the observed causal relationship, thereby enhancing the interpretability and translational application value of our research results.

## Methods

### Data sources

The ExWAS analysis included 35,680 eligible participants from the UK Biobank (UKB Application *No. 217841*), a population-based cohort comprising over 500,000 participants aged 40–69 years who completed standardized baseline assessments (including questionnaires, physical examinations, electronic health records and others) at 22 assessment centers with rigorous quality control protocols [18]. After excluding participants lacking neuroimaging data, those with baseline neurological disorders, and those with missing data on covariates, the final cohort was established with written informed consent and ethical approval from the North West Multi-centre Research Ethics Committee (MREC, https://www.ukbiobank.ac.uk/learn-more-about-uk-biobank/about-us/ethics) (Additional file 1: Fig. S1). Baseline time was defined as the dates of the participants’ first visits to the MRI assessment centers, coinciding with the collection of clinical data. Censored time was defined as the earliest date of disease diagnosis, death, or the last available date from the hospital or general practitioner, whichever occurred first. The study featured complete follow-up through March 2025 with a follow-up duration of over 10 years. BD was diagnosed and classified according to the International Classification of Diseases (ICD)-9 and ICD-10 codes. The specific codes include 296.0, 296.4, 296.5, 296.7, and 296.8 under ICD-9, as well as F31.0 through F31.9 under ICD-10.

We compared basic demographic and lifestyle characteristics between individuals with BD and non-BD controls. Among 502,629 UK Biobank participants, 1,258 individuals with ICD-defined BD were identified after excluding participants who had withdrawn consent and those with incomplete baseline demographic or lifestyle information (Additional file 1: Fig. S1). Subsequently, 2,516 non-BD controls were selected from the remaining UK Biobank participants using a 1:2 random sampling approach.

For the genetic analyses, summary statistics for BD were obtained from large-scale GWAS study. In these GWAS, BD cases were identified using cohort-specific clinical diagnostic procedures based on internationally accepted criteria, such as DSM-IV and ICD-based diagnoses, while controls were defined according to each cohort’s protocol. Specifically, summary statistics from a large-scale GWAS meta-analysis (41,917 cases and 371,549 controls) [19, 20] were used for the LDSC and primary MR analyses, while summary statistics from a GWAS conducted by PGC 2019 (20,352 cases and 31,358 controls) were used for validation MR analyses (https://www.med.unc.edu/pgc/) [21, 22]. The GWAS data for the 686 brain IDPs in this study were derived from neuroimaging analysis of 33,224 individuals of European ancestry in the UK Biobank, publicly available at https://open.win.ox.ac.uk/ukbiobank/big40/ [12, 23, 24]. Detailed information on the GWAS included in this study is provided in Additional file 2: Table S1.

In this study, a total of 686 brain IDPs were included for in-depth analysis, encompassing 329 structural IDPs and 357 diffusion IDPs. Spatially, these brain IDPs are widely distributed across various brain regions, specifically including 202 cortical IDPs, 127 subcortical IDPs, and 357 white matter tract IDPs. Regarding data processing, the UK Biobank employed systematic resolution to process dMRI data and utilized two complementary analytical methods—Tract-Based Spatial Statistics (TBSS) and probabilistic tractography analysis—to comprehensively assess brain microstructural characteristics. Among these, TBSS analysis, based on the brain atlas provided by Susumu Mori’s team at Johns Hopkins University, conducted detailed investigations into 48 white matter microstructure channels. Simultaneously, the study employed the brain atlas defined by AutoPtx to perform probabilistic tractography analysis on 27 white matter tracts [25, 26]. Based on previous studies [23, 27, 28], we obtained data on 11 key metrics, including fractional anisotropy (FA), mode of anisotropy (MO), mean diffusivity (MD), intracellular volume fraction (ICVF), orientation dispersion index (OD), isotropic or free water volume fraction (ISOVF), regional and tissue volume, cortical area, cortical thickness, regional and tissue intensity, and cortical gray-white matter contrast, which capture complementary aspects of brain macrostructure and microstructure. Detailed information on these metrics can be found in Additional file 2: Table S2. For further details on data quality control, preprocessing methods, and specific procedures, please refer to the official website of the UK Biobank.

### ExWAS analysis

To systematically conduct a data-driven exploration of observational associations between a comprehensive set of 686 brain IDPs and BD, we performed ExWAS analysis. The analysis utilized data from 35,680 eligible UK Biobank participants, who were randomly partitioned into equally-sized discovery and replication datasets to facilitate a robust internal validation process. Association analyses in both datasets were conducted using Firth’s penalized likelihood logistic regression, which is well suited for rare-event outcomes and reduces small-sample bias while improving model convergence under conditions of sparse data or complete/quasi-complete separation [29]. Three sequential models were fitted: (i) Model 0, an unadjusted, univariate model; (ii) Model 1, a minimally adjusted model correcting for age, sex, the first 10 genetic principal components, and genotyping array; and (iii) Model 2, a fully adjusted model including all covariates from Model 1 plus educational attainment, TDI, smoking status, and alcohol intake frequency. For each IDP, we computed the odds ratio (OR) and its 95% confidence interval (CI). To ensure the comparability of effect sizes, all continuous IDP variables were *z*-score standardized (mean = 0, Standard Deviation = 1), while the most common category served as the reference for categorical variables. IDPs that demonstrated nominal significance (*P* < 0.05) in the discovery dataset were carried forward as candidate associations for replication. In the replication dataset, the Benjamini-Hochberg FDR correction was applied to these candidate IDPs, and an association was considered positively replicated if it achieved FDR < 0.05 in the replication dataset with a consistent direction of effect relative to the discovery dataset.

### Genetic correlation analysis

We initially conducted genetic correlation analyses to screen for IDPs that are genetically associated with BD. LDSC is a method to quantify the contribution of true polygenic signal and confounding bias by examining the relationship between test statistics and linkage disequilibrium (LD), and can be applied to the estimation of heritability components, and the estimation of genetic correlations [13]. We performed genetic correlation analyses for each “IDP-BD” pair using LDSC based on GWAS statistical data for BD and 686 brain IDPs. The LDSC protocol can be found at https://github.com/bulik/ldsc. We used the LDSC *v.1.0.1* software in Python *v.2.7.18*. SNPs were screened using the HapMap-3 reference panel with minor allele frequency (MAF) > 1% for the 1000 Genomes European population.

### Two‑sample MR analysis

We included SNPs associated with each IDP as instrumental variables (IVs), with each SNP required to be: (1) strongly associated with the exposure factor independent of any potential confounders; and (2) capable of influencing the outcome variable exclusively through the exposure factor without direct effects on the outcome. In addition, other assumptions, including linearity and absence of statistical interactions, need to be met. For BD and each IDP, we first excluded SNPs with MAF < 1% and MAF close to 50% from the pooled GWAS data to avoid that these SNPs exert a critical impact on the genetic framework of this study [30]. SNPs with a genome-wide significance threshold of *P *< 5e-8 were included. Also, to ensure the independence of the IVs and to avoid misleading results, SNPs within the long LD region were also excluded (block window size = 1000 kb, *r*^*2*^ < 0.001) [31]. In the exposure GWAS, we calculated the *F* statistic to rate the strength of the SNPs to show the strength of the tool. SNPs with an *F* statistic greater than 10 could be taken into account for potential IVs, reducing the weak tool bias [32]. The *F* statistic was calculated as *F* = *R*^*2*^*(N-1-K) /[(1-R*^*2*^*)K]*, where *R*^*2*^ is the percentage of exposure variation attributable to genetic variation, *N* is the sample size, and *K* is the number of instrumental variables. We used the PhenoScanner to check for potential covariates, and SNPs significantly associated with confounders were excluded directly, including smoking behaviors, alcohol use disorder and education (Additional file 2: Table S3). All harmonized SNPs for each exposure-outcome pair in the initial and validation sets are archived in Additional file 2: Table S4.

We performed two-sample MR analyses using the TwoSampleMR R package (https://mrcieu.github.io/TwoSampleMR) in R *v. 4.1.2* to explore causality and direction between IDPs and BD. Forward MR analysis was performed with each IDP as exposure and BD as outcome. Exposure and outcome exchanges were subjected to reverse MR analysis. We used the inverse variance weighting (IVW) method of random-effects modeling for the primary MR analysis to assess the causal effect of exposure on outcomes [33]. Additionally, four complementary MR methods were used to enhance the reliability of MR analysis results, including: MR-Egger, weighted median, simple mode and weighted mode [34–36].

To further validate the results of the MR analysis, we conducted sensitivity analyses [37]. First, we used the MR-Egger regression intercept method and the MR Pleiotropy Residual Sum and Outlier (MR-PRESSO) global test to verify the presence of horizontal pleiotropy. If the intercept of the MR-Egger regression had a *P* greater than 0.05, it indicated that there was no horizontal pleiotropic effect. If outliers were detected, we then used the MR causal estimates recalculated by the MR-PRESSO method as the primary outcome, excluded outliers with *P* < 0.05, and reanalyzed the remaining SNPs using IVW analysis. Second, to complete the test for heterogeneity and ensure the robustness of the MR analysis, we used Cochran’s Q statistic. Next, we performed leave-one-out (LOO) sensitivity analyses, i.e., we excluded one SNP at a time and applied IVW random analysis of the remaining SNPs to assess whether causality was dominated by a particular SNP. To rule out the effects of idiosyncratic pleiotropy, systematic pleiotropy, and weak instrumental variables on causality, we similarly used the MR-Robust Adjusted Profile Score (MR.RAPS) technique [38]. Additionally, the GWAS summary statistics for BD used in the primary MR analysis were derived from a meta-analysis of multiple cohorts, including the UK Biobank, which may partially overlap with the UK Biobank neuroimaging cohort used for the GWAS of brain IDPs. To further evaluate the potential influence of sample overlap on MR estimates, a sensitivity analysis was performed using GWAS summary statistics for BD derived from the same meta-analysis with the UK Biobank cohort excluded [39]. All analyses in this study were mainly done using the “TwoSampleMR” and “MR-PRESSO” software packages in R *v. 4.1.2* software.

To enhance comprehensiveness, we performed a co-effects meta-analysis of the study results for both primary MR analyses and validation MR analyses using the “rmeta” R package. We used 0.05 as the statistical standard and adjusted the *P* value using the Bonferroni correction. If the *P* value was less than 0.05, the presence of causality was indicated, and for *P* less than 0.008 (0.05/6) suggests a significant association.

### Gene-based association analysis

To detect genes associated with specific brain IDPs and BD, we used the MAGMA method. Summary statistics for BD were obtained from PGC 2019 (20,352 cases and 31,358 controls, while GWAS data for the brain IDPs were derived from neuroimaging analyses of 33,224 individuals of European ancestry in the UK Biobank. MAGMA is a widely used tool for the analysis of genes and gene sets based on a multiple regression model [40]. We used MAGMA to transform GWAS-based associations at the SNP-level into associations at the gene-level to incorporate LD between variants and to find multivariate effects. We calibrated the LD structure against the LD reference panel based on the European-based 1000 Genomes Project. MAGMA can provide each gene with a separate *P* to assess its correlation with the disease. If there is a FDR < 0.05 in the MAGMA test, the gene is considered to be remarkably associated with the trait. According to the remarks provided by https://ctg.cncr.nl/software/magma, we defined this part of SNPs as SNPs located within a given gene.

### Pleiotropic Analysis under Composite Null Hypothesis (PLACO)

Pleiotropic Analysis under Composite Null Hypothesis was used by us to perform pleiotropic analysis [41]. We utilized the *P* values provided by the MAGMA analysis and converted them into *Z*-statistics. These converted *Z*-statistics were utilized to perform the analysis of pleiotropy under the composite null hypothesis. We divided the composite null hypothesis of pleiotropy into three null hypotheses: the gene is associated with the first disease but not with the second; the gene is associated with the second but not with the first; and the gene is not associated with either. The H^1^ hypothesis would be that the gene is associated with both. We corrected the *P* of PLACO using the FDR method. Genes with FDR < 0.05 were defined as pleiotropic genes associated with two traits.

### Functional analysis of pleiotropic genes

Following the identification of pleiotropic genes through PLACO, FUMA was employed to conduct differential expression analysis and gene-set enrichment analysis. The gene expression data were primarily sourced from GTEx *v8*, encompassing 54 distinct tissue types. To identify differentially expressed genes (DEGs), a two-sided t-test was applied to compare the expression levels of each gene against all others. Genes meeting the criteria of a Bonferroni-corrected *P* ≤ 0.05 and an absolute log fold change ≥ 0.58 were classified as DEGs for a specific tissue, indicating significant expression differences compared to other tissues. The direction of gene regulation—upregulation or downregulation—was determined by the sign of the t-score, with positive values indicating upregulation and negative values indicating downregulation.

Gene Ontology (GO) and Kyoto Encyclopedia of Genes and Genomes (KEGG) pathway enrichment analyses were further performed on pleiotropic genes identified by PLACO to explore the underlying biological mechanisms linking BD and IDPs. GO enrichment analysis was conducted across the three domains, including biological process, cellular component, and molecular function. All enrichment analyses were implemented using the R package “clusterProfiler”, and the results were visualized using the “ggplot2” and “enrichplot” packages. Statistical significance was defined as an adjusted *P* value (FDR) < 0.05.

### PPI network construction and significance evaluation

The PPI network was constructed and graphically represented utilizing Metascape in conjunction with Cytoscape software (*v. 3.10.2*). Metascape aggregated interaction data from multiple databases such as STRING, BioGrid, OmniPath, and InWeb_IM. The MCODE algorithm was employed to detect highly interconnected network clusters. Subsequent functional characterization of these clusters involved pathway enrichment and biological process analyses, with the three most statistically significant terms (based on *P*) being retained as representative functional annotations for each cluster.

### Spatiotemporal gene expression landscape across human brain development

Utilizing human mRNA sequencing data from the PsychENCODE consortium, we investigated the spatiotemporal expression patterns of pleiotropic genes shared between BD and subcortical brain volumes across the lifespan [42, 43]. The dataset encompasses multiple developmental stages (from embryonic week 8 to 40 years of adulthood) and nine distinct brain regions. Gene expression levels were quantified using Reads Per Kilobase of transcript per Million mapped reads (RPKM) values, followed by log₂ transformation and mean-centering normalization. For each brain tissue, we calculated mean gene expression values, with the average expression of shared genes representing the expression profile of each volumetric trait. Finally, we employed non-linear locally weighted regression (LOESS) to model expression trajectories, elucidating dynamic changes in gene expression patterns throughout different developmental periods.

## Results

### ExWAS identifies observational associations between IDPs and BD

Table 1 presents the demographic and lifestyle characteristics of the participants stratified by BD status. Compared with controls, participants with BD were younger, were more likely to be female and current smokers, and showed different patterns of alcohol consumption (*P* < 0.001). In terms of ethnicity, educational attainment, and socioeconomic status as measured by the Townsend Deprivation Index (TDI), no statistically significant between-group differences were observed.

**Table 1:** Characteristics of participants

Characteristic	BD cases (n = 1258)	Controls (n = 2516)	*P* value^a^
Age at MRI scan, years, mean (SD)	55.39 (7.87)	56.95 (8.06)	*<0.001*
Sex, n (%)	—	—	*0.004*
Female	735 (58.4%)	1344 (53.4%)	—
Male	523 (41.6%)	1172 (46.6%)	—
Ethnicity, n (%)	—	—	0.273
White	1198 (95.2%)	2373 (94.3%)	—
Non-White	60 (4.8%)	143 (5.7%)	—
Educational attainment, n (%)	—	—	0.0856
High (college/university or above)	643 (51.1%)	1027 (40.8%)	—
Intermediate (A/AS levels or equivalent)	165 (13.1%)	208 (8.3%)	—
Low (no qualification/GCSE/O-level)	450 (35.8%)	644 (25.6%)	—
Missing/unknown	0 (0.0%)	637 (25.3%)	—
Townsend Deprivation Index, quintiles, n (%)	—	—	0.181
Q1 (least deprived)	242 (19.2%)	516 (20.5%)	—
Q2–Q4	744 (59.1%)	1518 (60.3%)	—
Q5 (most deprived)	272 (21.6%)	482 (19.2%)	—
Smoking status, n (%)	—	—	*<*0.001
Never	657 (52.2%)	1307 (51.9%)	—
Former	390 (31.0%)	890 (35.4%)	—
Current	211 (16.8%)	306 (12.2%)	—
Missing/unknown	0 (0.0%)	13 (0.5%)	—
Alcohol intake frequency, n (%)	—	—	* <0.001*
Never/special occasions only	273 (21.7%)	554 (22.0%)	—
1–3 times/month	160 (12.7%)	255 (10.1%)	—
1–2 times/week	313 (24.9%)	665 (26.4%)	—
3–4 times/week	248 (19.7%)	541 (21.5%)	—
Daily or almost daily	264 (21.0%)	499 (19.8%)	—
Missing/unknown	0 (0.0%)	2 (0.1%)	—

*BD* bipolar disorder, *SD* standard deviation

^a^*P*-values were calculated using an independent-samples t-test for continuous variables and χ² (chi-squared) tests for categorical variables.

**Fig. 2:**
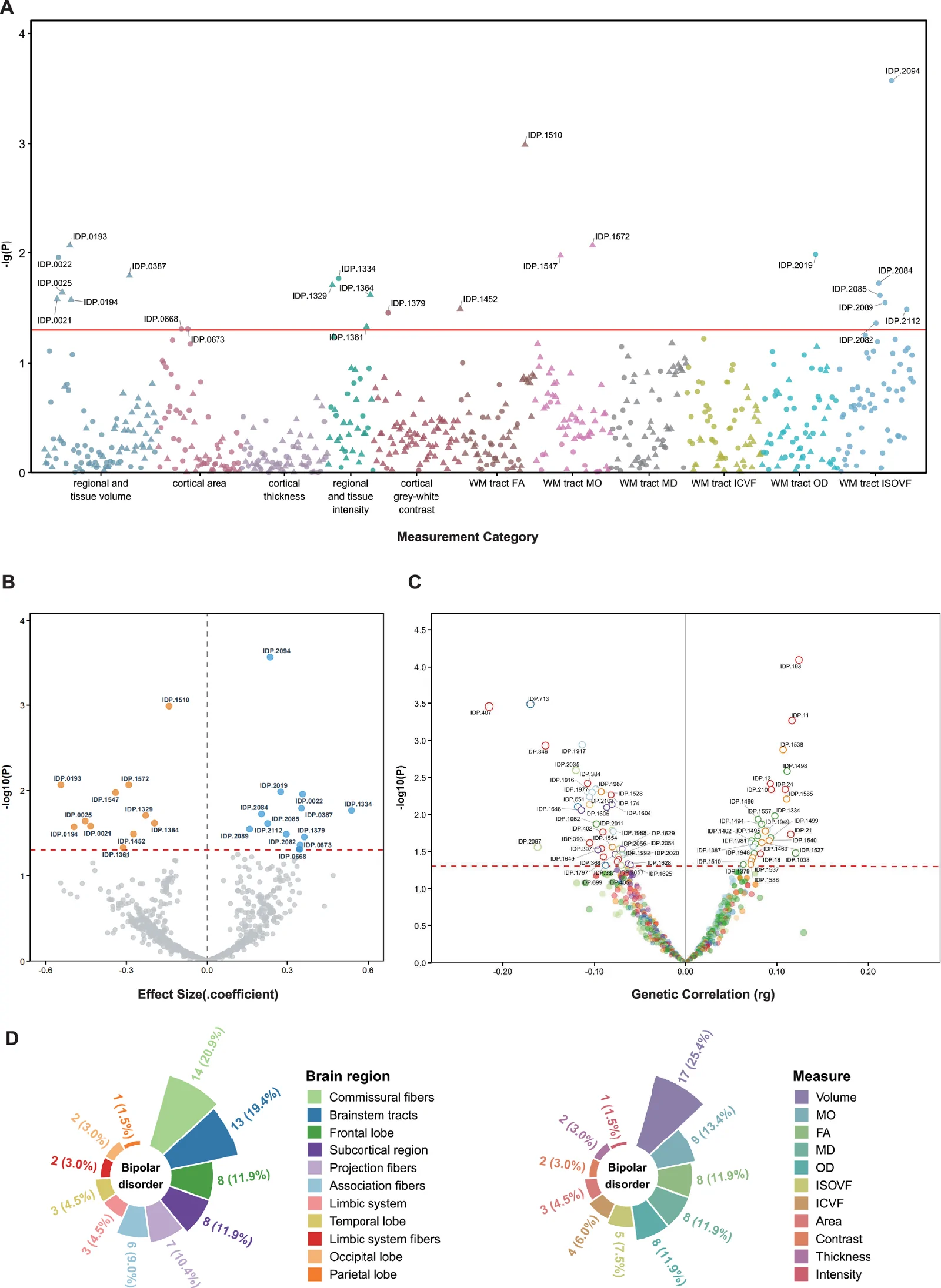
Exposome-Wide Association Study (ExWAS) and LDSC results of brain IDPs in BD. **A** Manhattan plot displaying the-log₁₀(*P*) values for associations between various IDPs and BD across different measurement categories . The red horizontal line indicates the significance threshold. **B** Volcano plot illustrating the effect sizes (β coefficients) and corresponding-log₁₀(*P*) values for IDP-BD associations. The dashed red line denotes the significance level. **C** Scatter plot of genetic correlations (rg) between IDPs and BD, with the dashed red line representing the threshold for significant genetic correlation. **D** Pie charts showing the distribution of BD-associated IDPs by brain region (left) and measurement type (right), with percentages indicating the proportion of IDPs in each category

To initiate our investigation with a hypothesis-free screen, we performed an ExWAS on 686 IDPs in the UK Biobank cohort. After correcting for multiple testing and validating the findings in the replication dataset, our analysis identified 10 IDPs that showed a robust and consistent observational association with BD status, defined by a FDR < 0.05 in replication sets (Fig. 2A-B; Additional file 2 Table S5). These validated IDPs spanned multiple domains, such as frontal lobe gray matter and commissural white matter tracts. For instance, positive associations were observed between the volume of the right lateral orbital frontal cortex (IDP.0387) and BD, and negative associations were observed between FA in the forceps major and tapetum (IDP.1510) and BD. These initial observational results provided a strong empirical basis for our subsequent genetic analyses.

### Genetic correlation analysis

LDSC was used to assess the extent to which the genetic associations were underpinned by shared genetic factors. We performed genetic correlation analyses for all 686 IDPs with BD. After correcting for multiple testing, we identified 67 IDPs that showed a significant genetic correlation with BD (nominal *P* < 0.05 threshold applied for screening purposes) (Fig. 2C), including IDP.0387 (volume of the right lateral orbital frontal cortex), IDP.1510 (FA in the forceps major), IDP.0713 (cortical surface area of the right frontal pole) and others. These genetically correlated IDPs were predominantly located in commissural fibers (20.9%) and brainstem tracts (19.4%). In terms of IDP subtypes, regional and tissue volume (17/67) and white matter tract mode of anisotropy (MO) (9/67) were the most represented categories (Fig. 2D). These results confirm a significant shared genetic architecture between specific brain structures and BD (Additional file 2: Table S6), providing us with more favorable genetic evidence for the final confirmation of the IDPs.

### The causal effects of brain IDPs on BD

We then performed MR analysis to explore the possible causal relationship and direction between BD and 67 IDPs. Through the two-sample MR analysis in the initial stage, we found that a total of 12 brain IDPs were causally related to the risk of BD. These included 3 frontal lobe IDPs, 4 commissural fibers IDPs, 1 subcortical region IDPs, 1 association fibers IDPs, 1 projection fibers IDPs, and 2 brainstem tracts IDPs. Their specific IDP names, SNP numbers, and ORs can be seen in Additional file 2: Table S7.

**Fig. 3:**
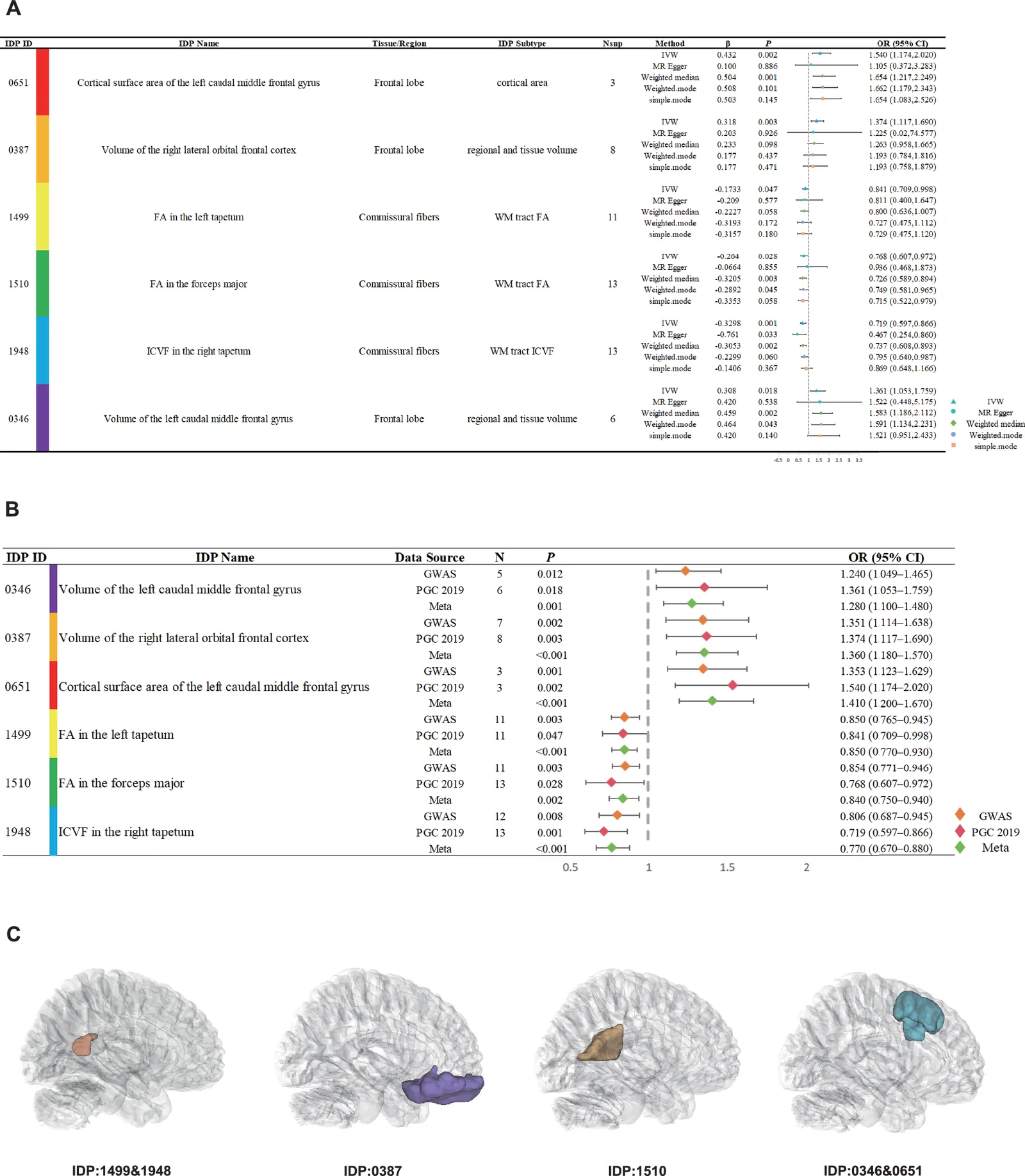
Mendelian randomization (MR) analysis results in the validation phase. **A** MR analysis summary table for brain IDPs associated with bipolar disorder, presenting details such as IDP ID, name, tissue/region, subtype, sample size (N), analytical methods (e.g., IVW, MR Egger), effect size (β), *P*, and odds ratio (OR) with 95% confidence interval (CI). **B** Forest plots illustrating the association of IDPs with bipolar disorder across different data sources, displaying *P* and OR with 95% CI. GWAS denotes the discovery dataset, PGC 2019 denotes the validation dataset, and Meta denotes the combined meta-analysis. **C** Three-dimensional brain renderings highlighting the anatomical localization of representative IDPs (IDP.1510, IDP.0387, IDP.1499 & 1948, IDP.0346 & 0651) implicated in bipolar disorder

For IDPs having significant causal relationships with BD, we performed the validation of the forward MR analysis. In the PGC consortium cohort, we successfully verified the causal effect of 6 IDPs on BD risk (Fig. 3A). More specifically, genetic determined IDP.0346 (volume of the left caudal middle frontal gyrus) (OR = 1.361, 95% CI: 1.053–1.759, *P* = 0.018), IDP.0387 (volume of the right lateral orbital frontal cortex) (OR = 1.374, 95% CI: 1.117–1.690, *P* = 0.003) and IDP.0651 (cortical surface area of the left caudal middle frontal gyrus) (OR = 1.540, 95% CI: 1.174–2.020, *P* = 0.002) could increase the risk of BD and was categorized as a risk factor for BD; while genetic determined IDP.1499 (FA in the left tapetum) (OR = 0.841, 95% CI: 0.709–0.998, *P* = 0.047), IDP.1510 (FA in the forceps major) (OR = 0.768, 95% CI: 0.607–0.972, *P* = 0.028), IDP.1948 (ICVF in the right tapetum) (OR = 0.719, 95% CI: 0.597–0.866, *P* = 0.001) was associated with an decrease in BD risk. For these six validated IDPs, the results of the IVW were consistent with the directions of the MR-Egger, Simple mode, Weighted Median and Weighted Mode in the initial and replication phases (Fig. 3A; Additional file 2: Table S8, Additional file 1: Figs. S2–S3).

The MR-Egger intercept test indicated no evidence of horizontal pleiotropy across all instrumental variables (*P* = 0.758 for the cortical surface area of left caudal middle frontal gyrus, *P* = 0.885 for the volume of right lateral orbitofrontal cortex, *P* = 0.630 for FA in the left tapetum, *P* = 0.886 for FA in the forceps major, *P* = 0.430 for ICVF in the right tapetum, and *P* = 0.811 for the left caudal middle frontal gyrus volume. Cochran’s *Q* test revealed no significant heterogeneity among SNPs (all *P* > 0.05), except for a nominal signal in IDP.1948 (ICVF of the right tapetum) (*P* = 0.001), which was also confirmed by the MR-PRESSO global test (*P* = 0.05). The results of the LOO analysis and funnel plots are presented in Additional file 1: Figs. S4–S5. Collectively, these analyses support the statistical stability and reliability of our MR results (Additional file 2: Table S9). Furthermore, sensitivity analyses using a UKB-excluded GWAS of BD showed that all 12 IDPs remained statistically significant, with consistent effect directions and comparable effect estimates relative to the primary analysis. These results suggest that the observed causal associations are unlikely to be driven by potential sample overlap (Additional file 2: Table S10).

Integrating data from two large-scale GWAS datasets, meta-analysis revealed statistically significant positive associations for three IDPs with increased BD risk (Fig. 3B): IDP.0346 (volume of the left caudal middle frontal gyrus) (OR = 1.280, 95% CI: 1.100–1.480; *P* = 0.001), IDP.0387 (volume of the right lateral orbital frontal cortex) (OR = 1.360, 95% CI: 1.180–1.570; *P* < 0.001), and IDP.0651 (cortical surface area of the left caudal middle frontal gyrus) (OR = 1.410, 95% CI: 1.200–1.670; *P* < 0.001). Conversely, three other IDPs demonstrated significant protective effects: IDP.1499 (FA in the left tapetum) (OR = 0.850, 95% CI: 0.770–0.930; *P* < 0.001), IDP.1510 (FA in the forceps major) (OR = 0.840, 95% CI: 0.750–0.940; *P* = 0.002), and IDP.1948 (ICVF in the right tapetum) (OR = 0.770, 95% CI: 0.670–0.880; *P* < 0.001). Both fixed-effects and random-effects models yielded consistent results, with no evidence of heterogeneity observed across studies (all *I²* = 0%). These robust findings identify distinct neuroimaging biomarkers that may serve as either risk or protective factors for BD, providing valuable insights for early identification and targeted intervention strategies (Additional file 1: Fig. S6).

### The causal effects of BD on brain IDPs

To further explore the causal relationship between BD and brain IDPs, we also performed a reverse MR analysis. In the preliminary MR analysis, a total of 6 IDPs showed causal associations with BD (Additional file 2: Tables S11–S13). However, none of these IDPs reached statistical significance in the replication analysis. Nevertheless, two other IDPs reached nominal significance in the replication analysis and showed consistent estimated directions across the MR-Egger, simple mode, weighted median and weighted models. Specifically, BD showed nominal associations with lower IDP.1510 (FA in the forceps major) (OR = 0.934, 95% CI: 0.880–0.991, *P* = 0.025), and higher IDP.1992 (OD in the left cerebral peduncle) (OR = 1.068, 95% CI: 1.000–1.141, *P* = 0.050). Multiple MR methods (Weighted Median, Weighted Mode, Simple Mode) showed consistent effect directions for both IDPs, although with wider confidence intervals.

Sensitivity analyses using MR-Egger regression revealed no significant pleiotropic bias (IDP.1510: *P* = 0.387; IDP.1992: *P* = 0.927). Heterogeneity tests were also non-significant (IDP.1510: *P* = 0.563; IDP.1992: *P* = 0.271), and MR-PRESSO detected no significant outliers (*P* > 0.05). MR-RAPS estimates further confirmed the effect for OD in the left cerebral peduncle (OR = 1.062, 95% CI: 1.008–1.118, *P* = 0.023).

### Potential shared genes between brain IDPs and BD

**Fig. 4:**
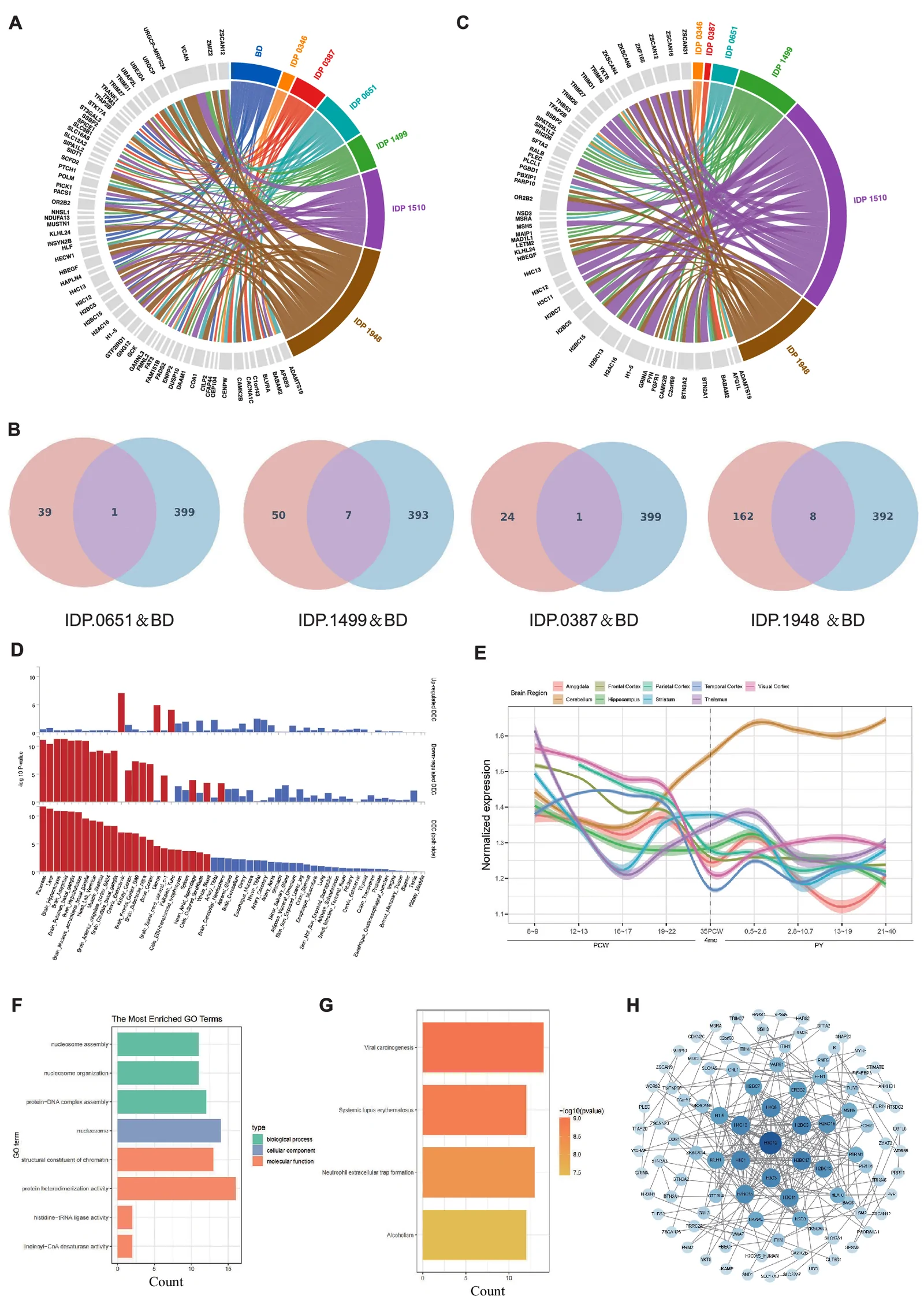
Multi-dimensional analyses of shared genes between brain IDPs and (BD. **A** Chord diagram: Displays associations between genes, BD, and six key IDPs (IDP.0346, volume of the left caudal middle frontal gyrus; IDP.0387, volume of the right lateral orbital frontal cortex; IDP.0651, cortical surface area of the left caudal middle frontal gyrus; IDP.1499, FA in the left tapetum; IDP.1948, ICVF in the right tapetum; IDP.1510, FA in the forceps major). **B** Venn diagrams: Illustrate the overlap of significant genes between BD and four IDPs (IDP.0651, IDP.1499, IDP.0387, IDP.1948), with numbers indicating shared gene counts. **C** Chord diagram: Shows pleiotropic gene associations with brain IDPs and BD identified via PLACO analysis. **D** Tissue enrichment analysis (FUMA, GTEx *v8*): Depicts differential expression significance of pleiotropic genes (shared between BD and six IDPs) across 54 tissue types; red bars signify higher significance. **E** Normalized expression dynamics: Descriptive trajectories of pleiotropic gene expression across multiple brain regions from post-conception weeks (PCW) to postnatal years (PY), highlighting temporal variation across developmental stages. **F** Gene Ontology (GO) enrichment: Highlights top enriched terms for pleiotropic genes, grouped by biological process, cellular component, and molecular function. **G** KEGG pathway enrichment: Displays significantly enriched pathways for pleiotropic genes. **H** Protein-protein interaction (PPI) network: Represents interactions of pleiotropic genes; node size corresponds to connectivity (larger nodes = more interactions)

We identified 5, 25, 40, 57, 170 and 14 genes significantly associated with IDP.0346 (volume of the left caudal middle frontal gyrus), IDP.0387 (volume of the right lateral orbital frontal cortex), IDP.0651 (cortical surface area of the left caudal middle frontal gyrus), IDP.1499 (FA in the left tapetum), IDP.1948 (ICVF in the right tapetum) and IDP.1510 (FA in the forceps major) by MAGMA analysis (FDR MAGMA < 0.05) separately. Also, 400 genes were identified as significantly associated with BD (FDR MAGMA < 0.05). We then examined gene-level overlap between BD and 6 IDPs. The number of shared genes with BD varied substantially: ICVF in the right tapetum (n = 8), FA in the left tapetum (n = 7), cortical surface area of the left caudal middle frontal gyrus (n = 1), and volume of the right lateral orbital frontal cortex (n = 1). The remaining 2 IDPs showed no genetic overlap with BD (Fig. 4A, B; Additional file 2: Table S14).

Next, PLACO was applied to identify pleiotropic genes shared between brain IDPs and BD. Of these, 4 genes were jointly associated with volume of the left caudal middle frontal gyrus and BD, 1 gene with volume of the right lateral orbital frontal cortex and BD, 9 genes with cortical surface area of the left caudal middle frontal gyrus and BD, 24 genes with FA in the left tapetum and BD, 33 genes with ICVF in the right tapetum and BD, and 143 genes with FA in the forceps major and BD (FDR PLACO < 0.05) (Fig. 4C; Additional file 2: Table S15).

### Tissue and functional enrichment analysis

The FUMA tissue enrichment analysis (GTEx *v8*, 54 tissue types) showed that genes shared between BD and 6 IDPs exhibited significant differential expression patterns across multiple tissues. Several tissues showed strong enrichment of DEGs after Bonferroni correction. Notably, brain-related tissues, including the frontal cortex, amygdala, and hippocampus, exhibited prominent DEG enrichment. Additionally, non-neural tissues such as the pancreas and liver were also enriched. The DEG sets were ranked by two-sided *P*-values to prioritize the most statistically significant associations (Fig. 4D). GO enrichment analysis of these pleiotropic genes revealed significant functional annotations across biological processes, cellular components, and molecular functions. The most prominently enriched terms included nucleosome assembly and nucleosome organization, nucleosome and structural constituent of chromatin, as well as protein heterodimerization activity, histidine–tRNA ligase activity, and linoleoyl–CoA desaturase activity (Fig. 4F). KEGG pathway analysis identified significant enrichment in four key pathways associated with these pleiotropic genes, including viral carcinogenesis, systemic lupus erythematosus, neutrophil extracellular trap formation, and alcoholism (Fig. 4G).

### PPI network reveals central role of histone proteins in BD-associated gene interactions

The PPI network analysis of pleiotropic genes reveals their interaction landscape at the protein level. In the network, nodes represent proteins and edges indicate known or predicted interactions between them. The overall structure of the network shows clear modularity, with a densely connected core region, suggesting that these central proteins may play critical regulatory roles. Among them, H3C12 exhibits the highest degree of connectivity and is located at the core of the network, indicating its potential key role in the biological processes related to BD. Other highly connected nodes include H2BC5, H3C11, H2BC15, H2AC16, H2BC13, H3C3, and H4C13, most of which are members of the histone family. This implies that the pathogenesis of BD may be closely linked to chromatin remodeling and epigenetic regulation. In addition, several non-histone proteins such as MLH1, MSH5, GTF2H4, and ERBB2 also show high connectivity, suggesting involvement in DNA repair, transcriptional regulation, and signal transduction pathways. Overall, this PPI network highlights the tight interactions among pleiotropic genes at the protein level, particularly a highly interconnected subnetwork centered around histone proteins, suggesting a potential role of epigenetic mechanisms in the development of BD. (Fig. 4H)

### Spatiotemporally divergent expression dynamics of shared genes

Using the spatiotemporal gene expression atlas of brain tissue, we analyzed the developmental dynamics of pleiotropic genes. As shown in Fig. 4E, distinct expression patterns were observed across regions. The cerebellum exhibited a unique trajectory, with relatively low expression during the Post-Conception Weeks (PCW) that begins to rise sharply around PCW 35 and remains elevated postnatally into adulthood. The thalamus showed a rapid decline in early fetal stages (PCW 8–13), a resurgence around PCW 18–22, followed by a gradual postnatal decrease, forming a fluctuating bimodal pattern. The visual cortex showed high expression in early fetal stages, followed by a slow decline and stabilization after birth, with a slight increase in adulthood. The striatum, hippocampus, and parietal cortex generally displayed declining expression during mid-to-late fetal development and maintained moderate, relatively stable levels after birth. The amygdala showed a gradual decrease during fetal stages, sustained low expression through infancy to adolescence, and a slight rise in adulthood. Overall, BD-related genes exhibited region-specific developmental trajectories, with notable differences occurring from late fetal development to early postnatal stages (from PCW 35 to approximately 2 years of age), suggesting this interval may represent a potentially relevant developmental period during which BD susceptibility genes exert key neurodevelopmental effects (Fig. 4E).

## Discussion

BD is a severe psychiatric condition whose neurobiological underpinnings remain incompletely understood. While decades of neuroimaging research have documented structural and functional brain abnormalities in BD, the cross-sectional nature of these studies has precluded causal inference [27]. To overcome this limitation, our study employed a systematic, epidemiological and genetic framework to deconstruct the complex brain IDPs-BD relationship. We began with an ExWAS analysis in the UK Biobank to map the landscape of brain-image associations. This provided an empirical foundation for a more targeted LDSC analysis, which confirmed significant genetic correlations between specific IDPs and BD. Finally, we leveraged large-scale GWAS data to conduct a bidirectional MR framework to formally test for causality. Sensitivity analysis and meta-analysis were also performed. This comprehensive pipeline provides robust epidemiological and genetic evidence for a causal role of specific frontal lobe and white matter phenotypes in BD risk, providing genetic support for the theory of neuroprogression [44]. Importantly, this framework facilitates prioritizing genetically supported imaging markers that may contribute to future risk stratification, early identification, and disease monitoring strategies in BD.

The finding of this study was the identification of a close relationship among IDP.1510 (FA in the forceps major), IDP.0387 (volume of the right lateral orbitofrontal cortex), and BD. FA, a DTI metric reflecting white matter microstructural integrity, is notably reduced in the forceps major of BD patients, suggesting impaired interhemispheric connectivity particularly in posterior cortical regions [45, 46]. This white matter abnormality may underlie disruptions in emotional regulation and integrative cognitive processing characteristic of BD. BD is highly heritable, with the heritability of liability estimated at approximately 59%, and it shares some genetic susceptibility with other mental disorders such as schizophrenia [47, 48]. Large-scale GWAS has identified 298 genomic loci that are significantly associated with BD on a genome-wide scale. Through fine mapping studies, the risk genes related to neuronal excitability and synaptic function have been further clarified. These genes are enriched in GABAergic interneurons and medium spiny neurons, which are crucial for cortical regulation and reward processing - these functions are closely linked to the orbitofrontal-limbic-striatal circuit implicated in emotional regulation [47, 49].

The orbitofrontal cortex, as an important component of the prefrontal cortex, plays a critical role in emotional processing, decision-making, and reward processing [50]. In patients with BD, neuroimaging studies consistently report reduced orbitofrontal cortex volume, and this structural abnormality may be associated with clinical manifestations commonly seen in BD patients, such as emotional instability and impulsive behaviors [51]. Studies have found that reduced volume of the right orbitofrontal cortex is significantly correlated with executive function deficits and emotional regulation difficulties in BD patients. Notably, this brain structural abnormality is observable not only during acute episodes but also persists during remission periods, suggesting it may represent a trait marker rather than a state-dependent manifestation of BD [52]. The abnormal neural circuits formed between the right orbitofrontal cortex and other brain regions (such as the amygdala, anterior cingulate cortex, and striatum) further exacerbate functional impairments in the emotional processing network of BD patients [53]. It is noteworthy that abnormal volume of the right orbitofrontal cortex is not a specific alteration to BD, but is also observed in schizophrenia, major depressive disorder, and other mental illnesses [54]. Emerging evidence suggests that several structural brain alterations observed in psychiatric disorders may reflect transdiagnostic neurobiological mechanisms rather than disorder-specific changes [55]. Therefore, the IDPs identified in this study may represent neurobiological pathways relevant to BD. Future cross-disorder research is necessary to clarify the extent to which these imaging markers are shared or distinct across major psychiatric conditions.

The forward genetic analysis provided compelling evidence that variations in specific frontal lobe structures are causally implicated in the development of BD. We found that genetically predicted increases in the volume and cortical surface area of the left caudal middle frontal gyrus, and in the volume of the right lateral orbital frontal cortex, significantly increased the risk of BD. The middle frontal gyrus is a key node in the frontoparietal network, which is central to executive functions such as cognitive control and emotional regulation—core domains of impairment in BD [44]. Prior work, including large-scale meta-analyses from the ENIGMA consortium, has consistently reported widespread cortical thickness and volume alterations in BD, particularly implicating frontal regions in the disorder’s pathophysiology [56]. Similarly, the lateral orbitofrontal cortex is critically involved in evaluating punishments and suppressing inappropriate actions, and its dysfunction has been directly linked to the emotional dysregulation that characterizes both manic and depressive episodes [57, 58]. By establishing a causal link, our study suggests that genetically-driven volumetric increases in these specific frontal regions may represent a primary neurobiological vulnerability that disrupts critical regulatory circuits and predisposes individuals to the onset of BD. From a translational perspective, these frontal lobe IDPs may serve as potential imaging markers for future risk stratification of BD.

Conversely, our analysis identified specific white matter characteristics that appear to be causally protective. Genetically predicted higher FA in the left tapetum and forceps major, and higher ICVF in the right tapetum, were associated with a significantly reduced risk of BD. The tapetum and forceps major are critical fiber bundles within the corpus callosum, the brain’s largest commissure, responsible for integrating complex information between the cerebral hemispheres. FA is a widely used proxy for the coherence and structural integrity of white matter tracts [59], while ICVF, derived from advanced diffusion models like neurite orientation dispersion and density imaging (NODDI), provides a more specific biological measure of neurite density and complexity [60]. A large body of literature, including recent mega-analyses, has documented widespread reductions in white matter integrity across major commissural, projection, and association tracts in individuals with BD, often interpreted as a core feature of the disorder’s pathophysiology [61]. Our findings reframe this narrative. They provide causal evidence that pre-existing, genetically determined robustness and density of these key interhemispheric tracts confer resilience against developing the disorder. This shifts the focus from purely pathogenic processes to include the neurobiological factors that promote and maintain mental health, opening new avenues for preventative strategies aimed at strengthening white matter connectivity.

A novel and clinically significant insight from our study is the evidence for a bidirectional relationship: while brain structure influences BD risk, our reverse MR analysis indicates that a BD is a causal risk factor for altering brain structure. This suggests a deleterious “illness scarring” effect, supporting a model in which an initial neurodevelopmental vulnerability is exacerbated by the disorder’s progression—a concept termed neuroprogression [62]. Challenging a static view of brain abnormalities in BD, these findings point to a vicious cycle wherein illness episodes may accelerate microstructural degradation, which could then worsen symptoms—a hypothesis corroborated by longitudinal imaging studies [63]. Consistent with this framework, neuroimaging research has reported that structural alterations in regions such as the frontal cortex, hippocampus, and limbic structures are associated with illness duration and episode recurrence in BD [56, 64, 65]. Recent evidence further indicates that functional connectome reorganization differs across distinct BD illness states (e.g., manic, depressive, mixed, and remission episodes), supporting the potential of neuroimaging phenotypes as biomarkers for capturing episodic manifestations and monitoring illness progression [66]. In addition, diffusion MRI studies have consistently reported widespread white matter microstructural abnormalities in individuals with BD. Reduced fractional anisotropy FA in callosal pathways has been observed in BD [61]. These findings align with our reverse MR result suggesting that genetic liability to BD may contribute to reduced FA in the forceps major, a posterior callosal fiber bundle arising primarily from the splenium of the corpus callosum. Furthermore, our reverse MR analysis revealed a causal increase in orientation dispersion (OD) within the left cerebral peduncle, offering a novel perspective on BD’s pathology. Existing NODDI studies in BD are limited and have primarily reported microstructural abnormalities in gray matter and limbic-related regions, including cortical areas, the posterior cingulate cortex, and hippocampal regions [67]. In contrast, OD alterations in the cerebral peduncle have rarely been described. Traditionally associated with motor function, the cerebral peduncle is now implicated as a target of the disease process. Increased OD reflects greater fanning or disorganization of white matter fibers, suggesting that genetic liability for BD leads to fiber disorganization in this region. This implies that BD’s pathophysiological impact extends beyond classic affective circuits and may involve axon damage or tissue remodeling driven by systemic processes such as neuroinflammation and oxidative stress—both increasingly recognized as core components of BD pathophysiology [44]. This finding opens new avenues for investigating the broader, systemic neurobiological effects of BD. Taken together, these bidirectional findings suggest that brain IDPs causally influenced by BD may serve as potential longitudinal markers of illness progression or treatment response. Nevertheless, additional clinical studies are necessary to validate their utility in monitoring disease progression.

Functional enrichment analyses provided additional insight into the biological processes underlying the associations between BD and the identified IDPs. GO enrichment highlighted pathways related to chromatin organization, including nucleosome assembly and chromatin structural components. These processes are central to the regulation of chromatin architecture and transcriptional activity. Increasing evidence indicates that epigenetic mechanisms, including chromatin remodeling and histone modification, regulate neuronal differentiation, synaptic plasticity, and brain development [68]. Dysregulation of these processes has been implicated in the pathophysiology of psychiatric disorders, including BD [69]. Large-scale epigenomic studies further suggest that genetic risk loci for psychiatric disorders are significantly enriched in regulatory elements active in the prefrontal cortex, underscoring the role of chromatin-mediated transcriptional regulation in this brain region [70]. Collectively, these findings suggest that chromatin-related regulatory processes may constitute a molecular mechanism underlying structural variability in frontal cortical regions implicated in BD. In addition, KEGG pathway analysis highlighted immune-related processes, particularly the neutrophil extracellular traps (NETs) formation pathway. Dysregulated NETs formation has been linked to excessive inflammatory signaling and vascular injury, potentially compromising neurovascular integrity and white matter microstructure [71]. Consistently, elevated neutrophil counts and increased neutrophil-to-lymphocyte ratios have been observed in individuals with BD [72]. These findings suggest that neutrophil-mediated inflammatory processes may contribute to structural brain alterations observed in BD, highlighting a potential target for clinical intervention.

Finally, we must acknowledge the limitations of our study, which also point toward clear directions for future research. First, our analyses were conducted in large European ancestry cohorts, which may limit the generalizability of our findings to other populations. Given the limited availability of GWAS datasets with comparable neuroimaging phenotypes in non-European populations, future cross-ancestry studies are needed to replicate and validate these findings across diverse ancestral groups. Second, the GWAS data for BD do not distinguish between subtypes (e.g., BD-I and BD-II), which are known to have clinical and potentially neuroanatomical differences. Third, although MR can strengthen causal inference, the results should be interpreted with caution because potential violations of instrumental variable assumptions, such as horizontal pleiotropy, cannot be completely ruled out. Fourth, the UKB imaging cohort primarily consists of middle-aged and older adults. This age distribution, together with potential confounding from psychotropic medication use, may introduce residual confounding despite statistical adjustment for age and related covariates. Moreover, BD definitions varied across analytical stages, potentially introducing heterogeneity. Furthermore, gene-level analysis results should be interpreted with caution and further validated when considering IDP–BD pairs with only a few shared genes. The spatiotemporal gene expression analysis was descriptive and did not include formal statistical comparisons across developmental stages; thus, the observed late fetal-to-early postnatal expression changes require further statistical and biological validation. Additionally, although our study integrated large-scale epidemiological, genetic, and multi-omics evidence, it did not include direct experimental biological validation. Therefore, the identified genes, pathways, and tissues should be interpreted with caution, and future functional studies are needed to validate their relevance to BD.

## Conclusions

In summary, this study systematically explored the complex relationship between brain structure and BD by integrating genetic and neuroimaging data. Through ExWAS, LDSC, and bidirectional MR analyses, we demonstrated that right lateral orbital frontal cortex volume and FA in the forceps major played vital roles in BD etiology. These findings provide a multi-scale framework that connects brain phenotypes to BD, offering novel insights into the neurobiological underpinnings of BD and emphasizing the necessity of early intervention strategies.

## Supplementary information

Supplementary Material 1: Additional file 1: Figures S1-S6.

Supplementary Material 2: Additional file 2: Tables S1-S15.

## Data Availability

Summary-level GWAS data analyzed in this study are publicly available. Detailed dataset links and accession identifiers are provided in the Methods and Supplementary Materials. Further processed data generated during this study are available from the corresponding author upon reasonable request. The data that support the ExWAS findings of this study are available from UK Biobank. Restrictions apply to the availability of these data, which were used under license for this study. Data are available (https:/www.ukbiobank.ac.uk) with the permission of UK Biobank.
