# Supplementary material for: Brain imaging-derived phenotypes influence risk of bipolar disorder: comprehensive exposome-wide association and wide-angled genetic analyses
Source: BMC Med. 2026 Aug 17;24:559. doi: 10.1186/s12916-026-05152-9 (PMC13660068; doi:10.1186/s12916-026-05152-9)
Supplement: Supplementary file 1 [file 12916_2026_5152_MOESM1_ESM.pdf]

**Additional file 1**
**Figures**

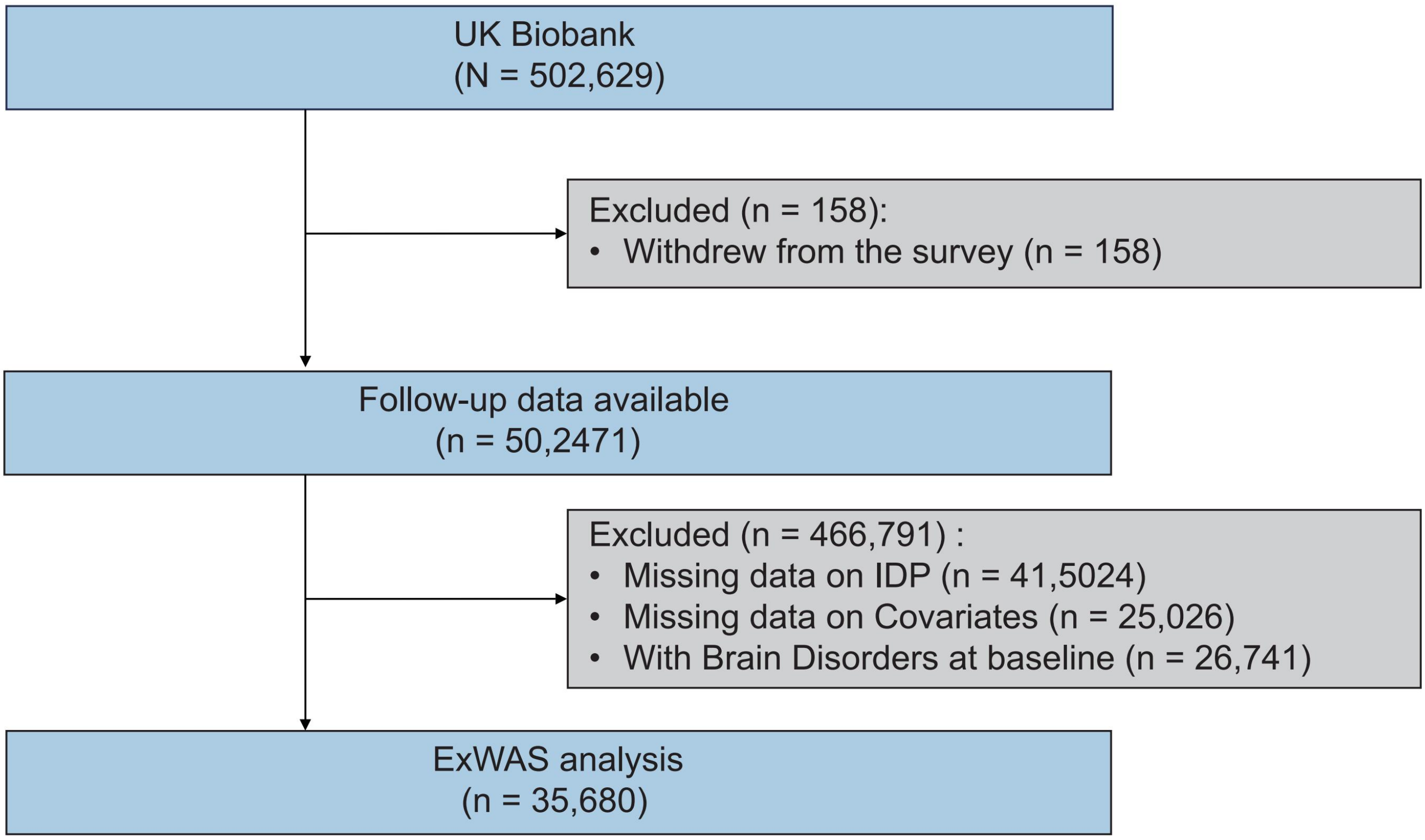


**Figure S1**: Flowchart of the UK Biobank participants included and excluded in ExWAS analysis.

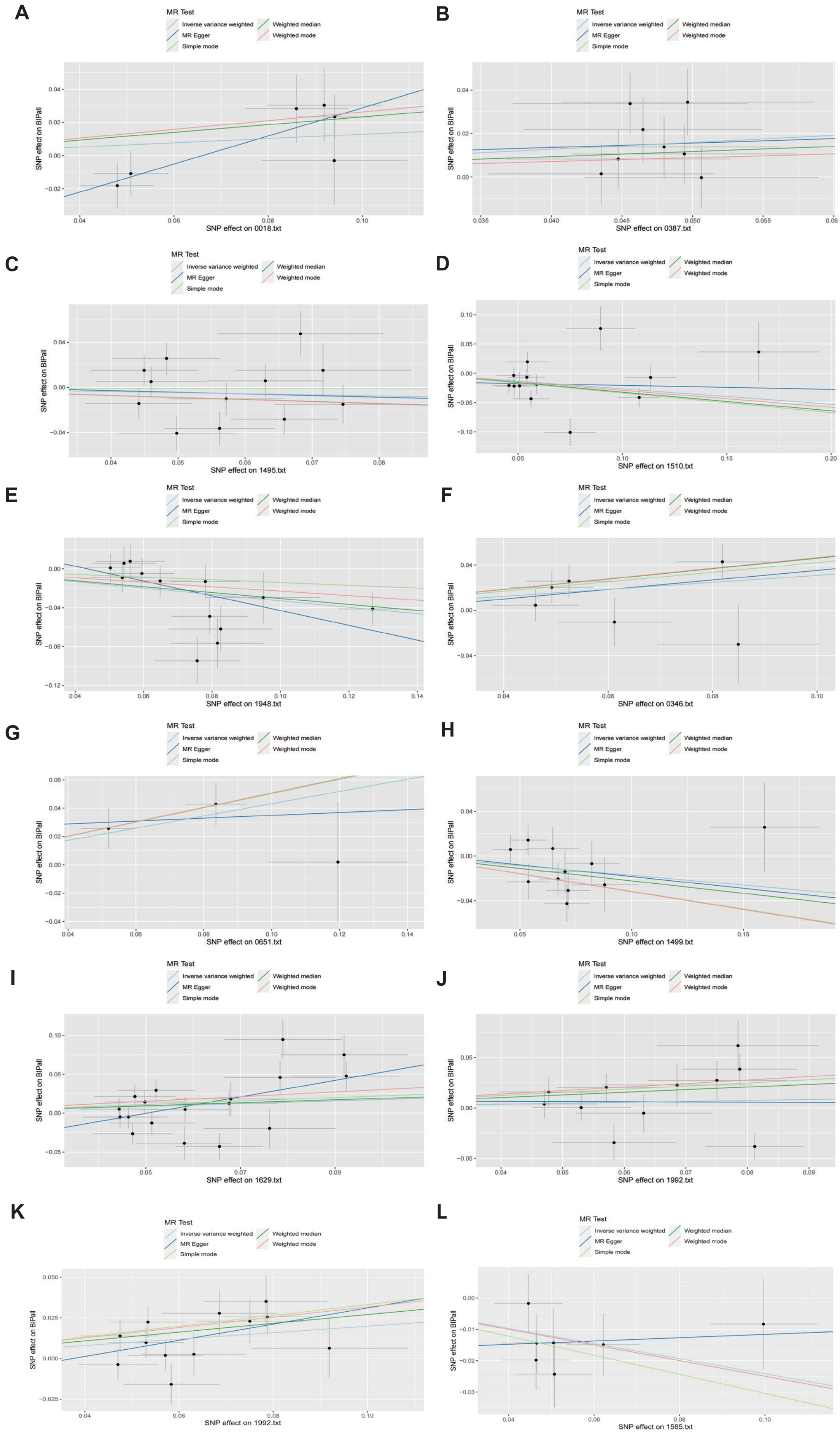


**Figure S2**: Scatter plots of MR analyses for associations between SNPs and IDPs.

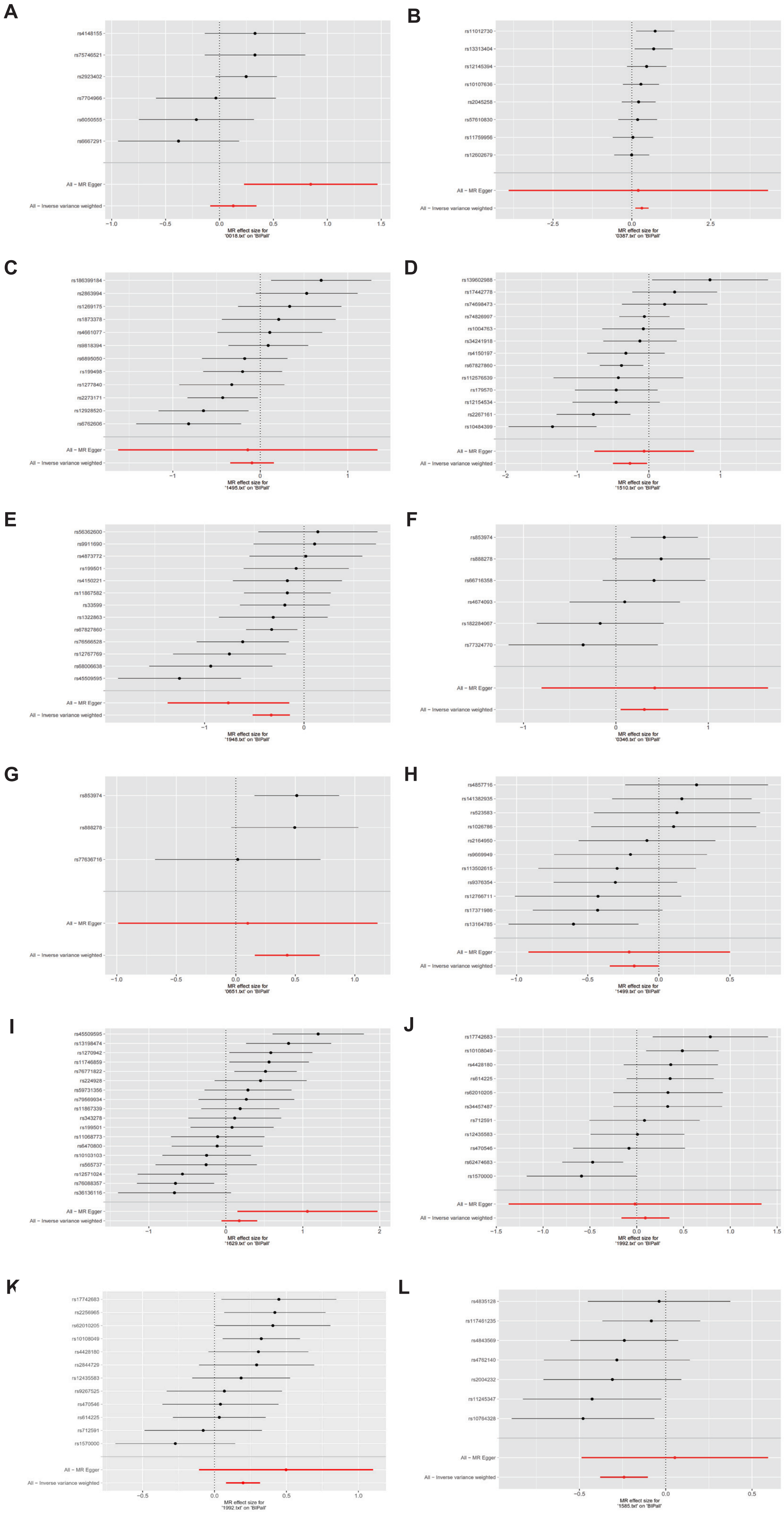


**Figure S3**: Forest plot of Mendelian randomization analysis assessing the causal effect of brain IDPs on the risk of BD.

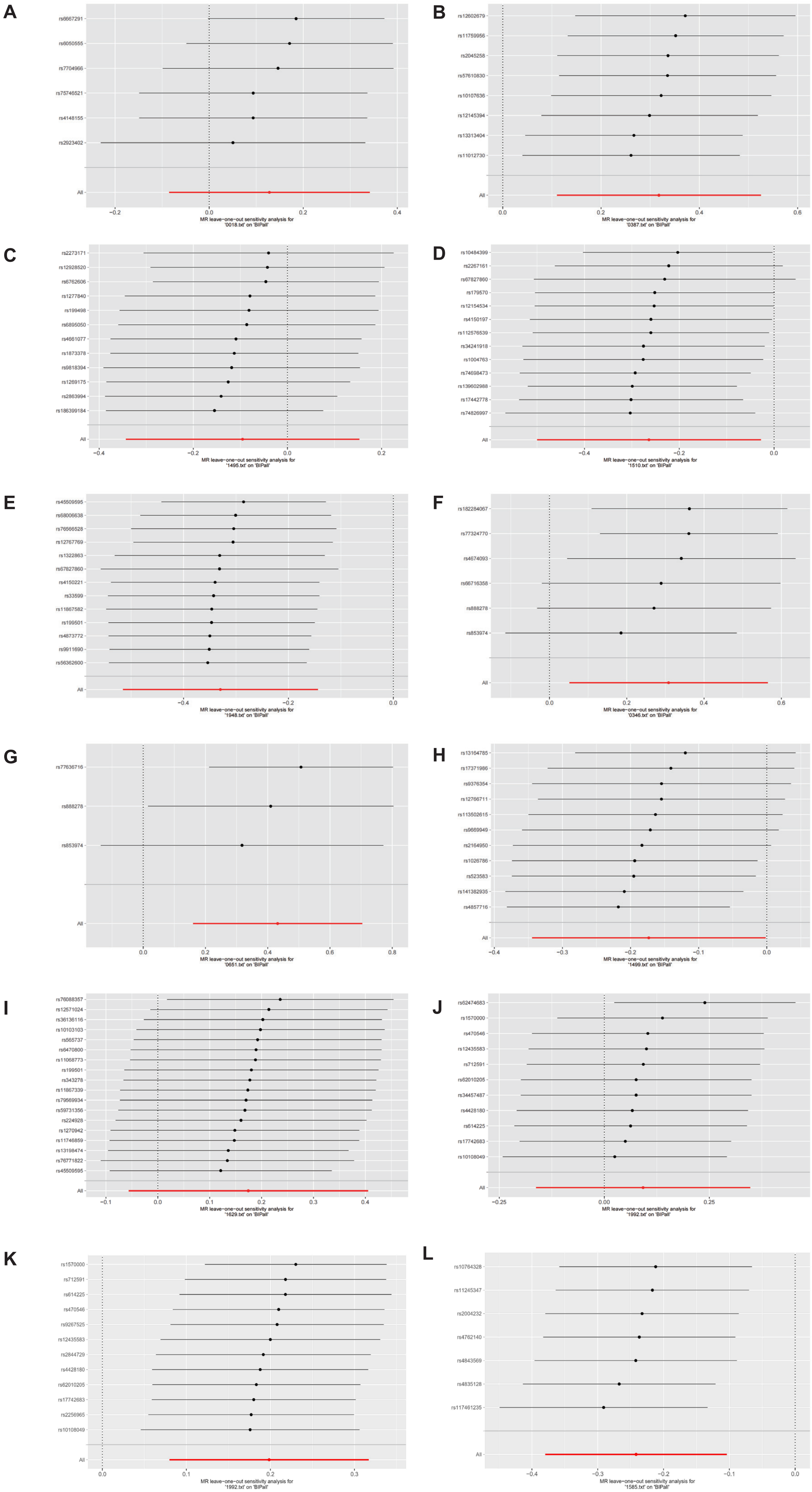


**Figure S4**: Forest plot of Mendelian randomization leave-one-out sensitivity analysis for the causal effect of brain IDPs on BD.

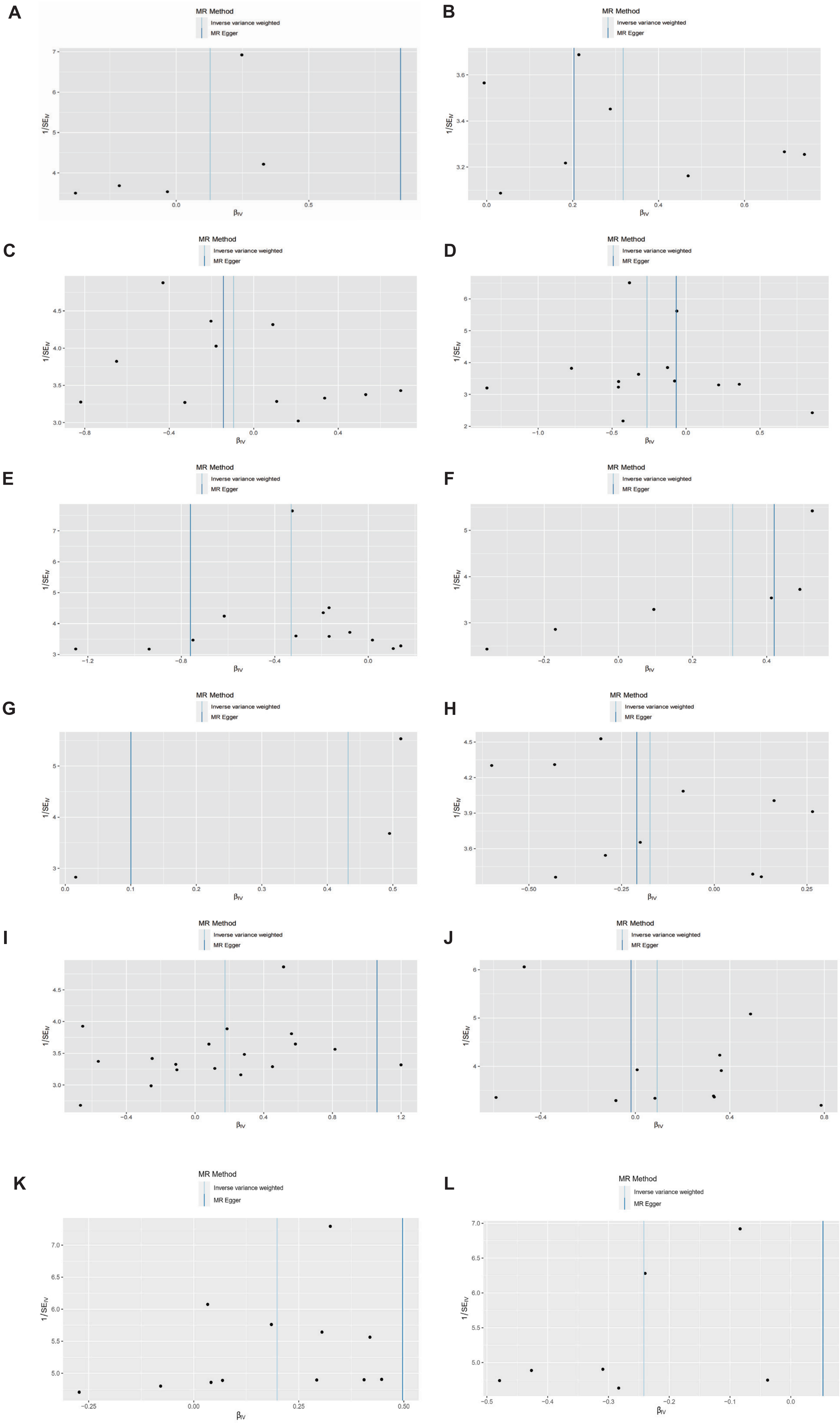


**Figure S5**: Funnel plots of the IVW and MR Egger methods in MR analysis for the causal effect of IDP on BD.

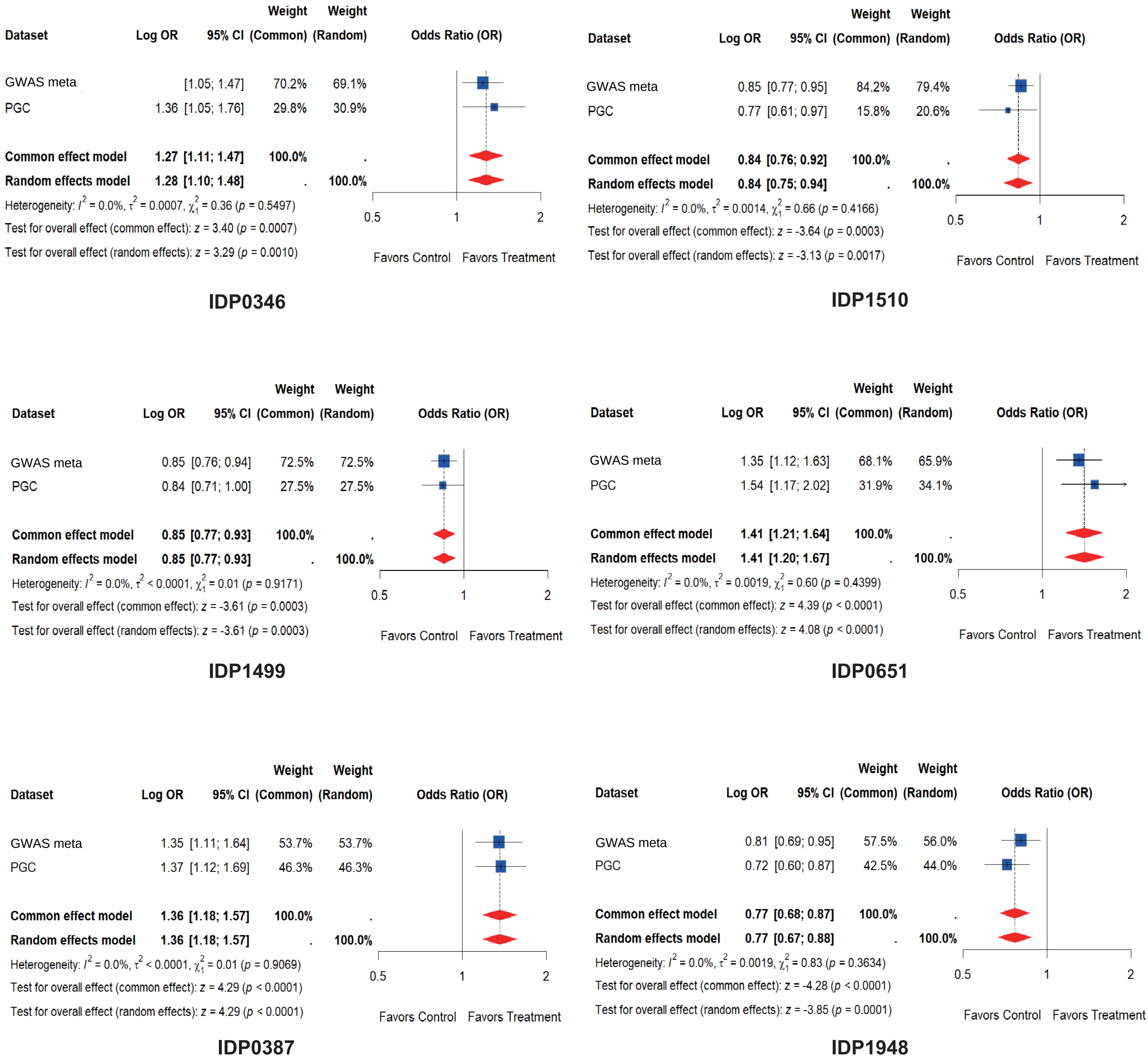


**Figure S6**: Forest plots presenting the association between six imaging-derived phenotypes (IDPs: IDP0346, IDP1510, IDP1499, IDP0651, IDP0387, IDP1948) and BD.
